# CONSENSUS: a Shiny application of dementia evaluation and reporting for the KU ADC longitudinal Clinical Cohort database

**DOI:** 10.1093/jamiaopen/ooab060

**Published:** 2021-08-02

**Authors:** Palash Sharma, Robert N Montgomery, Rasinio S Graves, Kayla Meyer, Suzanne L Hunt, Eric D Vidoni, Jonathan D Mahnken, Russell H Swerdlow, Jeffrey M Burns, Dinesh Pal Mudaranthakam

**Affiliations:** 1 Department of Biostatistics & Data Science, University of Kansas Medical Center, Kansas City, Kansas, USA; 2 University of California Davis Alzheimer’s Disease Center, Sacramento, California, USA; 3 University of Kansas Alzheimer’s Disease Center, Fairway, Kansas, USA

**Keywords:** Alzheimer disease, EDC, REDCap, R-studio, Shiny, consensus, data management

## Abstract

**Background:**

The University of Kansas Alzheimer’s Disease Center (KU ADC) maintains several large databases to track participant recruitment, enrollment, and capture various research-related activities. It is challenging to manage and coordinate all the research-related activities. One of the crucial activities involves generating a consensus diagnosis and communicating with participants and their primary care providers.

**Process:**

To effectively manage the cohort, the KU ADC utilizes a combination of open-source electronic data capture (EDC) (i.e. REDCap), along with other homegrown data management and analytic systems developed using R-studio and Shiny.

**Process evaluation:**

In this article, we describe the method and utility of the user-friendly dashboard that was developed for the rapid reporting of dementia evaluations which allows clinical researchers to summarize recruitment metrics, automatically generate letters to both participants and healthcare providers, which ultimately help optimize workflows.

**Conclusions:**

We believe this general framework would be beneficial to any institution that build reports and summarizing key metrics of their research from longitudinal databases.

## Introduction

Alzheimer’s disease (AD) is the most common neurodegenerative disorder that leads to a gradual decrease in cognitive abilities.^1^ From national vital statistics data, it is reported that AD is the sixth leading cause of death in the United States and a fifth leading cause among those age 65 and older.^2^ It is also estimated that approximately 5.4 million people have AD in the United States and during the last 30 years, mortality due to AD has increased gradually.^2^^,^^3^ In the United States, the age-adjusted death rate from AD increased by 39% from 2000 through 2010.^2^ One recent study reported an alarming change of the trend of life expectancy in multiple countries.^4^ For these reasons, the National Institutes of Health, specifically the National Institute on Aging, fosters AD research by providing federal funding to support Alzheimer’s disease centers (ADCs) at medical institutions throughout the United States. Each ADC and its affiliated researchers work to better understand the underlying risk factors of AD and improve patient care. This is done by conducting translational research and by collecting a substantial, longitudinal, standardized clinical, and neuropsychological dataset called the Uniform Data Set (UDS) developed and maintained by the National Alzheimer’s Coordinating Center (NACC).^5^ As one of more than 30 ADCs, the KU ADC, through clinical research and critical care, actively works to improve diagnoses, patient care, and educational resources for those affected by AD.

To enhance clinical activities and facilitate decision-making strategies, the KU ADC manages many research activities such as study enrollment, patient screening, and research data capture. These activities are exemplified in the KU ADC Clinical Cohort study, which is a prospective, longitudinal study, following around 400 participants, both with and without memory impairment. Each year, participants in the Clinical Cohort complete clinical and neuropsychological evaluations as part of the UDS. Organizing data capture and monitoring the cross-relationships in different research studies is an important function of the Data Management and Statistics (DMS) Core of the KU ADC. Additional uses for these cross-linked data include the generation of recruitment metrics for study sponsors and summary communications to participants and their physicians.

Historically, research data has been collected using the paper-based case report forms (CRFs) and then entered into a database to generate electronic records.^6^ Although the data collection process using CRFs is simple, error checking during entry is demanding and involves an additional validation step to ensure data safety and preserve the quality and integrity of the data.^6^ In recent years, electronic data capture (EDC) has become more prevalent with advances in hardware, software, and internet connectivity. With the broadening use of EDC, administrative bodies in the United States and Europe have provided guidelines to assure data safety, privacy, and data interchangeability.^7–10^ Thus, the EDC provides a platform to gather, manage, and store clinical research data more securely than ever. EDC allows simple access to the data, with security restrictions in place, which complies with regulatory standards, allows for real-time error checking, and helps to maintain data integrity.^6^^,^^11^ It is evident in the literature that EDC is more time-efficient for data capture and more time-saving when completing data validation while maintaining comparable error rates to paper-based approaches.^6^^,^^12^

The KU ADC uses an open-source EDC system for data capture which has accounted for improved efficiency in the speed of data transfer, consistent and periodic data reporting to the NACC. Digital storage provides reporting in real-time while maintaining data safety. It is prone to fewer transcription errors and thus improved overall data quality. Our EDC system provides a swift data management flow from initial contact with patients, to the final diagnosis stage, to uploading the data to the NACC database, fulfilling the requirements set by NACC. To further leverage the capabilities of an EDC-based system, we have developed and deployed web applications through which end users can easily find and summarize key variables and metrics to disseminate the clinical outcome and reports for the patients and physicians.

In this article, we aim to describe the development and functionality of a Shiny app to produce real-time reports for weekly diagnostic consensus meetings, a key activity involving integration of data from several sources and dissemination to several participants in multiple locations. This article attempts to summarize our entire consensus process of the KU ADC from data collection to data management and reporting. Specifically, we advocate for the idea of using open-source software and how the automation process improves efficiency based on user type. Furthermore, we discuss how the consensus Shiny app helps facilitate sending automated correspondence to both participants and their primary care physicians (PCPs) aiding ongoing engagement and retention efforts.

## Materials and methods

### Data collection

The KU ADC Clinical Cohort is a longitudinal study involving the collection of annual clinical and neuropsychological assessments, for participants with and without dementia.^13–17^ Participants enrolled in the KU ADC Clinical Cohort receive the required NACC^5^^,^^18^ UDS assessments, along with several other items collected for KU-specific research questions and to aid with operational components of the study. These longitudinal questionnaires and instruments are captured during the participants’ initial visit and subsequent follow-up visits, administered by highly trained research personnel, clinicians, and psychometrists. Each participant is accompanied by a family member or close friend as a collateral source for their annual clinical evaluation where information about the participant is captured (functional, behavioral, physiological, etc.). A research coordinator gathers and updates demographic information, medical history, family history, and medication usage, and administers surveys related to the participant's daily functioning and mood. The clinician performs a clinical interview, accompanied by a general neurological examination to complete a clinical dementia rating scale (CDR).^14^ Data are directly entered into the REDCap^19^ system. A neuropsychological test battery is also administered by a trained psychometrist following a standardized testing method.^13^^,^^14^ These test scores and summary of the neuropsychological battery follow NACC guidelines to satisfy the standard UDS 3.0 requirements. Handling of UDS data is described in our prior work.^20^ For diagnostic consensus reporting, we mostly utilize UDS 3.0 data from the cognitive test and CDR visits. More detailed recruitment methods and protocols were reported under these papers.^13^^,^^17^^,^^21^

### Data management and security

Utilizing open-source software has gained momentum in the research community due to the cost benefits, availability, and capabilities of working functionality across platforms. Study data are collected and managed through REDCap, a secure web-based software platform designed to support data capture for research studies, providing (1) an intuitive interface for validated data capture; (2) audit trails for tracking data manipulation and export procedures; (3) automated export procedures for seamless data downloads to common statistical packages; and (4) procedures for data integration and interoperability with external sources.^19^^,^^22^ A variety of open-source software was used to create the Shiny dashboard for conducting weekly consensus meetings. Data cleaning and reporting were managed through R,^23^ R studio,^24^ R markdown,^25^ and Shiny.^26^ R is an open-source programming language software for statistical computing and supported by R Foundation for Statistical Computing. R has versatile flexibility including custom user packages, graphical devices, import/export capabilities, and reporting (Knitr), among other capabilities. Primarily, we use R studio for data transformation, report generation. R Studio is also an open-source IDE (Integrated Development Environment) software for R and can be deployed in any operating system. The advantage of using R Studio is it can run on a server that enables multiple users to access the tools simultaneously, and when paired with the Shiny package can seamlessly develop a web-based dashboard. HTML (Hypertext Markup Language) and pdf reports used in diagnostic consensus meetings were generated by the R Markdown package from R library. Shiny is an R package used to build an interactive Web application and dashboard. We used the Shiny package which is hosted on a dedicated server to build and host real-time analytics and consensus reporting for our longitudinal Clinical Cohort study data.

A primary concern using open source software such as R, Python, etc. compared with other proprietary software, is to make sure that data privacy and any vulnerabilities are addressed appropriately.^27^ The balance between ease of access for relevant researchers and protecting a patient’s data must always lean in favor of the security of the data. The free version of Shiny does not provide a way to secure access to apps through a login screen. Instead of relying on pre-built login templates for R, our team integrated the Shiny app with KUMC’s Central Authentication Service (CAS) system. Consequently, we understand the structure of the code and its functionality and are confident that it works as intended in our environment. It also grants a group of users to access the shiny app with the single sign-on credential facilitated by the University of Kansas medical center (KUMC). Similarly, REDCap is also linked to the KUMC CAS system to serve as an extra layer of protection. All programs written by the programmers, coding of variables, outputs are stored in the secured server hosted by the DMS Core. External users or collaborators can submit a written request for permission to access the shared drive, Shiny app server, or any REDCap project associated with the consensus cohort database.

### Process

**Figure 1.**
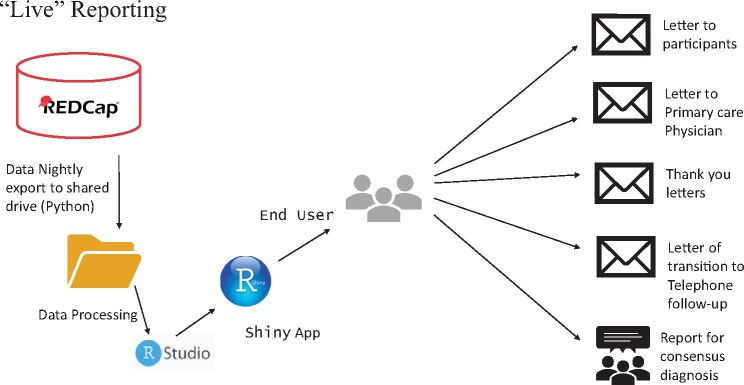
An overview of the dataflow of the consensus reporting using the Shiny app.

The KU ADC employs multiple projects in REDCap to capture various phases of the recruitment process. Initial research participation inquiries and study participation records were captured in our Participant Relations and Outreach Management Tool (PROMPT database).^21^ Clinical Cohort participant evaluations and tracking were performed in a separate database. A third database was used to track all past and ongoing human subjects research studies. A sketch of the process and workflow is displayed in Figure 1.

**Figure 2.**
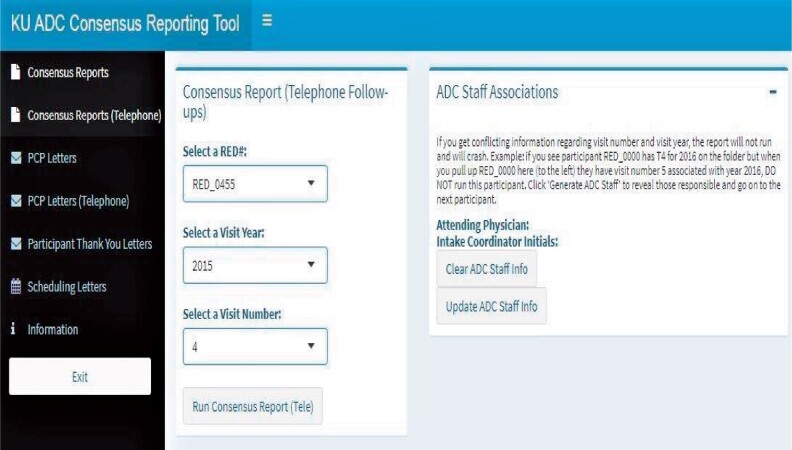
An overview of the diagnostic consensus app utilizing Shiny Dashboard. Visible is the tab for creating letters to participant’s primary care physician.

Data exports to the secure server were automated and implemented via a task scheduler called as cron job. The cron job executes a series of python code, which pulls down the exports listed from a bootstrap project. The bootstrap project is a REDCap project that lists all the relevant information (API key, fields to exports, filename to use, and project information) so that the python code can create the exports. The python code creates requests for each export listed in the bootstrap project and calls to the REDCap API. Next, the code generates the requested CSV files. Finally, a shell script moves the generated CSV files securely to the desired server location. Once all the data exports and transfers are completed, a series of R scripts are utilized to join all the datasets that come from various projects and creates a final dataset that is available for loading into the Shiny app for diagnostic consensus reports and writing letters. Figure 2 provides an overview of the Shiny app user interface.

**Figure 3.**
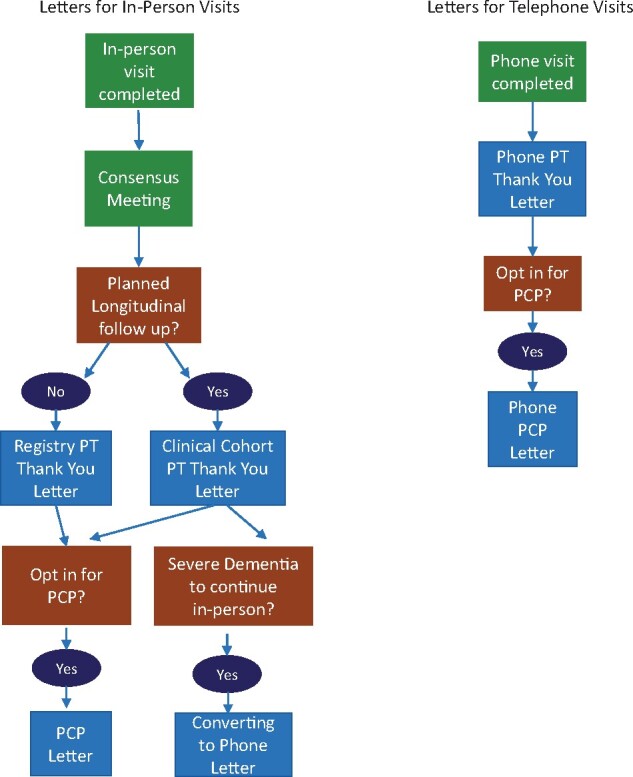
Flow diagram of in-person and follow-up visits with different functionality.

The user interface is user-friendly and does not require knowledge of any programming language. Users can generate a participant’s consensus report (usually in the Markdown format/HTML/pdf) by browsing and navigating the Shiny dashboard tab. Once the appropriate unique identifier is selected, code will run on the backend and display the reports. We incorporated multiple features into our Shiny app based on the need for the project and reporting metrics. Furthermore, this application helps to send automated preformatted letters to both Clinical Cohort participants and their PCPs. The flowchart in Figure 3 assists in visualizing how in-person and telephone follow-up visits work with different functionality.

Every week a group of KU ADC clinicians, coordinators, and psychometrists meet for a diagnostic consensus conference to review participant cases, with a goal of consensus determination of cognitive change and diagnostic etiology. To facilitate this meeting, we use the Shiny app to generate diagnostic consensus reports (Supplementary Material S1) that aggregate and display cognitive testing, study partner comments, and the general impression and observations of the clinician. All relevant and necessary information is compiled into the consensus reports for ease of access and simplicity, streamlining the consensus conference. Via a Shiny Dashboard interface, one person manages display of the diagnostic consensus report on each individual for display to all meeting attendees. Through this interface, the user is not allowed to input the incorrect combination of the patient’s unique ID number, visit year, etc. The dashboard produces the standard reports along with the cognitive scores for each participant visit. Aggregation of these values across multiple REDCap events for a single participant, even if possible, would be difficult to decipher using standard REDCap reporting tools. Furthermore, it also displays the information on the KU ADC clinician, psychometrist, and intake coordinator who administered the CDR, current and prior neuropsychological cognitive tests and standardize comparison values, and collected demographic information, respectively. Additionally, this app can generate consensus reports for the participants who have reached the stages of dementia where they cannot continue in-person visits and instead are followed through telephone contact. However, in this case, there are no cognitive scores available and the reports may or may not have a participant rationale, depending on the functional ability of the participant. Custom coding allows for flexibility of reporting in these situations.

Some participants seek to share their results with their primary care provider. The consensus app can generate multiple versions of a PCP letter (Supplementary Material S2). This letter includes the physician’s name, street address, city, state, zip code, and final impression from the attending ADC clinician. This app does not generate PCP letters if the participant has opted out of this service. Based upon the participant consent, the consensus app determines whether a PCP letter can be created and looks for the presence of additional variables needed to complete the request, namely physician name and address. If the general structure is validated, selecting an available participant’s ID will produce the physician’s address along with the final impression from the attending physician or interviewer. The app pulls this information into a standardized letter template and saves a separate file with the PCP address, facilitating printing of both the letter and mailing envelopes. The same mechanism applies to telephone follow-up PCP letters. Like the PCP letter, this Shiny app can be used to send thank you letters (Supplementary Material S3) to the participants. If the participant’s street address, city, state, and zip code are not filled out correctly, the end-user will not be given the option to generate a letter. The “Thank You” letter to the participants includes individualized feedback to the participant. Additionally, it allows selecting whether the participants are part of the KU ADC single evaluation Registry Study, or if part of the longitudinal Clinical Cohort. Depending on the selection, the app will select the appropriate letter template, ensuring the correct language is used, minimizing end-user confusion. This Shiny interface also can generate scheduling reminder letters for the participants with expected longitudinal follow-up in the KU ADC Clinical Cohort. As shown in Figure 2, selecting a month from the Scheduling letters tab will reveal the number of regular in-person Cohort and telephone follow-up anniversaries are expected for that month. Running the Generate letters tab from the subsection of the Scheduling letters tab will produce letters for the listed participants. However, if someone had the following criteria such as screen failures, initial evaluations only, discontinued, deceased, minimal contact, or Registry only, this app will not produce a scheduling letter for those participants, as continued follow-up is not expected.

### Process evaluation

In this section, we illustrate some key performance metrics of this dashboard compared with a manual procedure. The diagnostic consensus Shiny app is run once a week, generally building reports for 5–10 participants. The time to create one consensus report based on user type and methods is shown in Table 1. An advanced user has at least 5 years of experience with a background in R and R-Studio programing and had been solely in charge of running the Consensus reports weekly for over 3 years. The novice user had worked at the KU ADC for under 1 year, had little experience with R and R Studio programing, and no experience running Consensus reports. Reports created manually require the user to click through the database, copying data from 5 different forms and pasting that data into the report. Multiple items are collected from each form. Data are collected from each of the participant’s completed visits with a maximum of 9 possible visits. A stopwatch was used to record the amount of time required to create reports. We created similar reports for multiple times (5 trials) depending on reporting methods and user types to measure the average reporting time. Three clinical core members of our KUADC cohort helped us to gather the data. From Table 1, we can see that creating one consensus report manually required an average of 556 s (CI: 447.69–664.31 s), i.e. 9.27 min for an advanced staff member. In contrast, the same process takes approximately 1208.6 s (about 20.15 min) for a novice user. Similarly, creating one consensus report using the automated Shiny app process takes about 70.2 s (about 1.17 min) for the advanced user and about 140.8 s (CI: 129.09–152.51 s) i.e. approximately 2.35 min for a novice user. When averaging advanced and novice users, the automated program reduced reporting time by 88.0%.

**Table 1.** Time to create one consensus report by user type and methods

User type	Reporting time (s)				Mean percentage decrease
	Manual time Mean (SD)	CI	Automated time Mean (SD)	CI	
Advanced	556 (55.26)	447.69–664.31	70.2 (3.96)	62.43–77.97	87.4%
Novice	1208.6 (38.90)	1132.35–1284.85	140.8 (5.97)	129.09–152.51	88.4%

Time reported in seconds; CI: Confidence Interval; SD: Standard deviation.

Looking over the past 5 years, an average of 5 participants are reviewed at the diagnostic consensus conference each week, with the maximum being 24 participants and the minimum being 1 participant. With an average of 5 participants needing reports created each week, it would take for an advanced user about 46.3 min to manually create 5 reports, versus 5.8 min by using the automated Shiny app system, saving even the most advanced staff user 40.5 min weekly. Thus, the Shiny-based reporting tools vastly reduce the time to generate reports. These time savings do not include the generation of PCP, Thank You, or Scheduling letters for participants, potentially saving even more time for users. Additional time is saved when the app generates PCP, Thank You, and Scheduling letters for participants. Also, as reported previously,^13^ before the implementation of the EDC platform (October 27, 2012), the median days required to have a consensus conference from the first evaluation was 31 days, whereas it is now reduced to 17 days. Similarly, using a paper-based approach (before October 27, 2012), was required 211.3 days from the first visit to individual record completion and NACC submission as compared to 95.1 days with this automated reporting tool.

## Discussion

In clinical and translation research, designing and implementing dashboard-based data integration and analytic visualization has gained popularity.^28–32^ Application-based infrastructure helps clinicians better to inform the patient’s behavior and medical history and guide them to make better informed diagnostic decisions.^28^^,^^29^ To the best of our knowledge, the consensus app is one of the first open source-based application systems designed for rapid reporting of dementia evaluation in research. Exploiting modern computational and programming power, this framework is adaptable for other clinical research-based reporting systems.

This report emphasizes leveraging computational power from a custom-designed Shiny app and simple user interface to promote rapid communication between research clinician and participant. We describe an R Shiny-based, open-source dashboard framework for generating reporting and correspondence. This app uses REDCap for data collection, R packages for data management, standardization of the data, and outputs standard HTML/pdf files for flexible use. This interactive web application has some attractive features to aid researchers in getting better insight into the progression of AD of the individual patient and helps inform pertinent longitudinal and follow-up information. Also, this app helps researchers improve screening time for trials. Participants can also benefit from this process by receiving their feedback quicker through the “Thank You” letter and rapid communication with their PCP. It has an intuitive graphical user interface. It is accessible via the internet within the KUMC network and can also be used through the web browser without installation. This allows researchers to aggregate visit data, retrieving required information and data quickly through this program, instead of pulling the records from the volume of paper files.

Adaptation of an open source-based framework has limitations too. There is always a concern about utilizing such user-friendly web-based apps for clinical research data evaluation and implementation over commercial counterparts. Furthermore, individuals need to have appropriate experience to use technologies and need proper training and logistics to enter data. A lack of integration and transferability of the Shiny application to other database management platforms such as Qualtrics, Castor EDC, ClinCapture, etc. might limit the applicability of the app. Another concern is the privacy and security of data management over the internet and for that reason, technical support needs more than community support. Finally, it requires time and extensive programming skills to develop such sophisticated systems, and maintenance is sometimes costly. Open-source software packages always update their system/package without any warranty, which might lead to unexpected errors. Thus, it is required to continuously monitor and update the new R packages and IDE versions of the software if available. Overall, based on our clinical team’s feedback, after the implementation of the Shiny app and EDC platform, both the time and cost for managing this large longitudinal cohort are meaningfully reduced.

## Conclusions

In conclusion, our consensus reporting Shiny app plays a vital role in KU ADC operations. It is expected that sending preformatted letters of appreciation, scheduling reminders, and minimal visit feedback to participants helps to acknowledge the patient’s role in our KU ADC cohort, which ultimately will help with participant retention. Another benefit of this application is by automating physician notification letters, the participants will receive feedback from highly skilled and trained dementia experts faster, thus the quality of the physician–patient experience will improve. Adaptation of such open-source system is widely applicable in other healthcare areas such as to visualize and track COVID data,^33^^,^^34^ infection management^35^ etc. Although the potential benefits of computer-based system in medical research areas, paper based reporting still used in parallel in many sectors.^36–39^ However, we envision that the implementation of the Shiny-based consensus-reporting system, in a broader spectrum, will enhance the research experience and efficiency of the overall assessment in various medical sectors.

## Funding

This research was supported by NIH grant P30 AG035982 through the National Institute on Aging.

## Author contributions

PS, RNM, DPM, KM, and SLH contributed to the overall planning and writing of the manuscript. RSG, EDV, RNM, and PS contributes to the development of the App. All authors have contributed to the writing and review of the final manuscript. All authors approved their contributions and the final draft of the manuscript.

## Ethics approval and consent to participate

The University of Kansas Medical center approved the study. Informed consent was obtained from each study participant.

## SUPPLEMENTARY MATERIAL

Supplementary material is available at *Journal of the American Medical Informatics Association* online.

## Data availability

Code and data related to Shiny App is the proprietary property of the KU ADC. Sharing of the code and related information is available upon reasonable request pending upon the approvals of IRB and HIPPA consent.

## CONFLICT OF INTEREST

The authors have no conflicts of interest to declare.

## Supplementary Material

ooab060_Supplementary_Data
