# Supplementary material for: CONSENSUS: a Shiny application of dementia evaluation and reporting for the KU ADC longitudinal Clinical Cohort database
Source: JAMIA Open. 2021 Aug 2;4(3):ooab060. doi: 10.1093/jamiaopen/ooab060 (PMC8327371; doi:10.1093/jamiaopen/ooab060)
Supplement: ooab060_Supplementary_Data [file ooab060_supplementary_data.zip › Supplementary materials 1, Consensus Report.pdf]

JAH - TEST_9999

KU ALZHEIMER'S DISEASE CENTER
The University of Kansas Medical Center

Date of Evaluation: December 01, 2020 by Jane Clinician; Visit: 2
RED Number: TEST_9999
Initials: JAH
DoB/Age: 1946-07-01 / 74 years old.
Sex: Female
Occupation: Teacher, Retired 2012, Normal Reasons
Education: 16
GDS Score: 2
Study Partner: Husband

Overview: 74 year old woman presents with husband for T2 evaluation. Overall health is stable. No recent changes. First noticed memory problems about 2 years ago. She's having trouble with short term and immediate recall. Only behavioral change is mild irritability over the last 6-8 months.

Clinical Dementia Rating Summary
Memory: CDR 0.5
Informant Rationale: Short term memory issues over the past few years, on the verge of being consistent. She's misplacing items more often and repeating herself. She has a hard time keeping track of appointments. She usually remembers an important recent events but may forget pertinent details. Recall is good for remote events.
Participant Rationale:Participant reports about 1 year course of inconsistent short term memory loss. Did not recall 1 week story at all. Only partially correct on 1 month story. 5/5 on immediate JB phrase. Autobiographic history correct other than when she retired. 0/5 on delayed JB phrase.

Orientation: CDR 1
Informant Rationale: She has difficulty keeping track of the date and day of the week. Some difficulty with time relationships. She's never been very punctual. She can navigate familiar places.
Participant Rationale: One day off on day of the week, otherwise fully oriented.

Judgement and Problem Solving: CDR 0.5
Informant Rationale: Problem solving ability is fair. Some loss in managing finances. She occasionally forgets to pay a bill. She would not be able to handle an emergency efficiently. She usually understands situations and is usually socially appropriate.
Participant Rationale: 4/4 on abstractions. 2/3 on calculations. Gave up on serial 3's. Correct answer on find a friend. Appears to have good insight.

Community Affairs: CDR 0
Informant Rationale: She stopped working for normal reasons. She is still active in the community, driving and navigating to familiar places without problem. Never great at navigating in unfamiliar places and may be asking husband to drive more often. A casual observer would not think that she is ill.

Home and Hobbies: CDR 0.5
Informant Rationale: She manages her routine chores as well as ever. Continues to clean and do her own laundry. She continues to sew, but with some loss in ability as she more frequently has to undo and repeat her work.

Personal Care: CDR 0
Informant Rationale: Independent without prompts.

Global CDR: 0.5

Neuro Examination:
No abnormal neurological findings

Objective Testing Results:
Largely incorrect on recall of events in last week.
Somewhat correct on recall of events in last month.
0/5 on delayed recall of the John Brown Phrase.
Correctly judged 2 similarity questions
Correctly judged 2 difference questions
Correctly answered 2 of 3 calculation questions
Correctly answered the judgement question

Single Clinician Impression: 74 year old woman returns for T2 evaluation. Approx 2 year history of gradually progressive problems with short term memory, orientation, and executive function. She remains functionally independent. Neuro exam was unremarkable. There were a few notable episodes of forgetfulness during the visit. CDR box score is 2.5. Global CDR is 0.5 indicating questionable impairment. Presentation suggestive of amnestic MCI.

NACC Designation: amnestic MCI with the additional following deficits: executive function

Cognitive Tests

RED Number: TEST_9999
Participant: JAH
Education: 16
GDS Score: 2
Tester: John Doe for visit 2

Cognitive Testing

Cognitive Testing Raw Values

Cognitive Test	Dec 01 2019	Dec 01 2020
MoCA Total Raw	23	19
MMSE	26	20
Craft Story 21 Immediate (Paraph.)**	11	6
Craft Story 21 Delayed (Paraph.)**	0	1
Free Recall 1	4	3
Free Recall 2	6	4
Free Recall 3	5	5
Cued Recall 1	8	11
Cued Recall 2	8	10
Cued Recall 3	11	9
Benson Complex Figure (Copy)	16	14
Benson Complex Figure (Recall)	5	3
MINT Total **	31	30
Category Animals	15	17
Category Vegetables	13	13
Verbal: F-Words Correct	9	8
Verbal: L-Words Correct	10	7
Verbal: F and L-Words Correct	19	15
Trail-Making A	32	40
Trail-Making B	76	111
Block Design	29	29
Digit Symbol Substitution	38	39
Stroop (Color)	61	49
Stroop (Word)	78	77
Stroop (Interference)	24	17
Number Span Forward	6	7
Number Span Backward	5	2
Letter Number Sequence	8	0

** - Prior to 24 March, 2015; Craft Stories = Logical and Delayed Logical Memory; MINT Total = Boston Naming Test

Number of Sub Tests in the Impaired Range:
December 01, 2020: Three or more scores are abnormal or lower than expected
December 01, 2019: Three or more scores are abnormal or lower than expected
