# Supplementary material for: CONSENSUS: a Shiny application of dementia evaluation and reporting for the KU ADC longitudinal Clinical Cohort database
Source: JAMIA Open. 2021 Aug 2;4(3):ooab060. doi: 10.1093/jamiaopen/ooab060 (PMC8327371; doi:10.1093/jamiaopen/ooab060)
Supplement: ooab060_Supplementary_Data [file ooab060_supplementary_data.zip › Supplementary materials 3, Cohort Participant Thank you letter.pdf]

KU ALZHEIMER'S DISEASE CENTER
The University of Kansas Medical Center

Date of evaluation: December 1, 2020

Dear Ms. Hawk,

Thank you for your participation in the KU Alzheimer's Disease Center Clinical Cohort. The Clinical Cohort is a long-term study focused on how to maintain a healthy brain and slow changes in memory and thinking that can occur as one ages. This project is part of a broader national effort and the information we collect is shared in a national database (after any identifying information is removed, such as name and date of birth). Thus, you are contributing to the national effort to better understand and fight the rising problem of Alzheimer's disease.

Results from your participation

Much of the information collected is research-specific and difficult to interpret on a personal level. We strive to share as much information with you as we can. Our clinicians have provided you with feedback during your clinical assessment. Below are additional results that you may find interesting. Feel free to share these results with your physician.

- Your Mini Mental Status Exam score was 20 out of 30 possible points. On average, people without memory problems score 29 on this assessment. Scores below 24/30 are generally considered to indicate cognitive impairment.

What's Next?

- We will continue to assess your memory and thinking every year. Our office will be contacting you in 9-10 months to schedule you for next year's evaluations.

Please consider attending our annual appreciation breakfast where we are able to provide you with updates concerning our program and research efforts.

We greatly appreciate your participation. Our work is a partnership with you in the fight to prevent Alzheimer's disease. If you have any questions, please contact our office at (913) 588-0555, or at kuamp@kumc.edu.

Sincerely,

Jeffrey M. Burns, M.D., M.S.
Edward H. Hashinger Professor of Neurology
Associate Director, KU Alzheimer's Disease Center
Department of Neurology
University of Kansas Medical Center
