# Supplementary material for: CONSENSUS: a Shiny application of dementia evaluation and reporting for the KU ADC longitudinal Clinical Cohort database
Source: JAMIA Open. 2021 Aug 2;4(3):ooab060. doi: 10.1093/jamiaopen/ooab060 (PMC8327371; doi:10.1093/jamiaopen/ooab060)
Supplement: ooab060_Supplementary_Data [file Supplementary materials 2, PCP Letter.pdf › Supplementary materials 2, PCP Letter.pdf]

KU ALZHEIMER'S DISEASE CENTER
The University of Kansas Medical Center

December 31, 2020

Re: Hawk, Jay A.
DOB: 1946-07-01

Dear Dr. Example,

I had the pleasure of seeing your patient, Ms. Hawk, for a research evaluation at the University of Kansas Alzheimer's Disease Center. This study enrolls people with and without memory problems. Participants have assessments of their memory and thinking to support ongoing research on the brain, aging, memory loss, and Alzheimer's disease.

Your patient has asked us to forward you information from their evaluation. Below is a summary of our clinical research assessment and physical examination.

**IMPRESSION**:
74-year-old woman returns for T2 evaluation. Approximately 2-year history of gradually progressive problems with short term memory, orientation, and executive function. She remains functionally independent. Neuro exam was unremarkable. There were a few notable episodes of forgetfulness during the visit. CDR box score is 2.5. Global CDR is 0.5 indicating questionable impairment. Presentation suggestive of amnestic MCI.

If you have any questions regarding your patient and our research assessment, please call (913) 588-0555.

Additionally, we have attached information about one of our clinical support initiatives. The Community Care Network supports Primary Care Providers in bringing a patient empowerment approach to individuals newly diagnosed through dementia specific social work navigators. If you would like to know more about this initiative, you can contact Michelle Niedens at 913.945.7310 or cniedens2@kumc.edu.

Sincerely,

Jane Clinician
